# Dynamic Changes in the Extracellular Matrix in Primary, Metastatic, and Recurrent Ovarian Cancers

**DOI:** 10.3390/cells11233769

**Published:** 2022-11-25

**Authors:** Arkadiusz Gertych, Ann E. Walts, Keyi Cheng, Manyun Liu, Joshi John, Jenny Lester, Beth Y. Karlan, Sandra Orsulic

**Affiliations:** 1 Department of Pathology and Laboratory Medicine, Cedars-Sinai Medical Center, Los Angeles, CA 90048, USA; 2 Department of Surgery, Cedars-Sinai Medical Center, Los Angeles, CA 90048, USA; 3 Faculty of Biomedical Engineering, Silesian University of Technology, 44-100 Zabrze, Poland; 4 Department of Mathematics, University of California Los Angeles, Los Angeles, CA 90095, USA; 5 Jiann-Ping Hsu College of Public Health, Georgia Southern University, Statesboro, GA 30458, USA; 6 Department of Veterans Affairs, Greater Los Angeles Healthcare System, Los Angeles, CA 90095, USA; 7 Department of Obstetrics and Gynecology, David Geffen School of Medicine, University of California Los Angeles, Los Angeles, CA 90095, USA; 8 Jonsson Comprehensive Cancer Center, University of California Los Angeles, Los Angeles, CA 90095, USA

**Keywords:** computational imaging, COL11A1, ECM, CAF, collagen, extracellular matrix, carcinoma-associated fibroblasts, high-grade serous ovarian cancer, tumor microenvironment

## Abstract

Cancer-associated fibroblasts (CAFs) and their extracellular matrix are active participants in cancer progression. While it is known that functionally different subpopulations of CAFs co-exist in ovarian cancer, it is unclear whether certain CAF subsets are enriched during metastatic progression and/or chemotherapy. Using computational image analyses of patient-matched primary high-grade serous ovarian carcinomas, synchronous pre-chemotherapy metastases, and metachronous post-chemotherapy metastases from 42 patients, we documented the dynamic spatiotemporal changes in the extracellular matrix, fibroblasts, epithelial cells, immune cells, and CAF subsets expressing different extracellular matrix components. Among the different CAF subsets, COL11A1^+^ CAFs were associated with linearized collagen fibers and exhibited the greatest enrichment in pre- and post-chemotherapy metastases compared to matched primary tumors. Although pre- and post-chemotherapy metastases were associated with increased CD8^+^ T cell infiltration, the infiltrate was not always evenly distributed between the stroma and cancer cells, leading to an increased frequency of the immune-excluded phenotype where the majority of CD8^+^ T cells are present in the tumor stroma but absent from the tumor parenchyma. Overall, most of the differences in the tumor microenvironment were observed between primary tumors and metastases, while fewer differences were observed between pre- and post-treatment metastases. These data suggest that the tumor microenvironment is largely determined by the primary vs. metastatic location of the tumor while chemotherapy does not have a significant impact on the host microenvironment.

## 1. Introduction

Despite substantial progress in the molecular understanding of cancer, several cancers commonly associated with desmoplastic stroma, such as pancreatic, ovarian, colorectal, breast, and lung cancers, remain difficult to treat with conventional therapies. Most therapeutic approaches have focused on epithelial tumor cells and their genetic alterations. However, tumor response to and/or the beneficial effects of personalized therapies targeting specific mutations in these cancers are typically temporary due to genomic instability and the rapid proliferation of resistant clones. Increasingly, normalization of the tumor microenvironment is thought to explain the efficacy observed with recent immunotherapies and some targeted agents. Identification of additional molecular events and cellular factors whose disruption might undermine tumor progression could lead to more successful anti-cancer therapies.

Cancer-associated fibroblasts (CAFs) are common in solid malignancies and their presence is associated with poor clinical outcomes (reviewed in [1]). CAFs secrete a dense extracellular matrix (ECM) that traps immune cells thus physically restricting their access to tumor islets [2,3,4]. In mouse tumor models, it has been observed that T cells are enriched in the CAF-secreted ECM while fewer T cells are seen in contact with cancer cells [2,3,4]. Immunostaining and real-time imaging of T cell distribution in viable slices of human tumors have revealed that fiber alignment and the density of ECM control T-cell migration [5]. The mechanisms by which CAF-secreted ECM might limit T-cell access to tumor islets are not clear but could result from a steric barrier of dense fibers within the stroma and/or increased interstitial fluid pressure within the tumor.

Although CAFs and immune cells are viewed as promising therapeutic targets, significant challenges remain in developing adequate tools to modify the stromal environment in cancer. Studies to assign specific functions to distinct types of stromal cells have been confounded by a poor understanding of stromal cell heterogeneity and a lack of effective targeted approaches. Several therapeutic approaches targeting stromal cells, including endothelial cells, hematopoietic cells, and CAFs, have been used with variable success in ovarian cancer patients. Anti-angiogenic agents, such as bevacizumab, effectively prolong progression-free, but not overall, survival [6]. Immunotherapies with antagonists to CTLA-4 [7], PD-1 [8], and PD-L1 [9] have yielded remarkable results, including complete responses in chemoresistant cancers and long-term remissions in melanoma and lung cancer [10], but only a small subset of patients respond to immunotherapy [10]. A clinical trial of anti-PD-1 therapy in melanoma revealed that innately resistant tumors upregulate the stromal genes involved in epithelial-mesenchymal transition, extracellular matrix remodeling, and wound healing [11], suggesting a possible role for CAFs in the immune escape. However, methods to effectively target CAFs without subjecting normal fibroblasts and stem cells to toxicity are still in the early stages of development.

High-grade serous ovarian carcinoma (HGSOC) is the most common and most lethal subtype of ovarian cancer. Standard treatment for HGSOC combines surgical cytoreduction with platinum-based chemotherapy. Typically, the treatment initially achieves remission; however, cancer recurs in the vast majority of women. Patients with the recurrent disease might continue to respond to additional cycles of platinum-based chemotherapy but most will ultimately develop platinum resistance. An analysis of human ovarian cancer samples demonstrated that the balance between CAFs and CD8^+^ T cells profoundly affects chemotherapeutic sensitivity [12]. While immune cell distribution has been extensively studied in primary tumors (reviewed [13]), less is known about the distribution of immune cell subsets during tumor progression and chemotherapy resistance. To elucidate changes in the tumor microenvironment during ovarian cancer progression, we investigated ECM structure, percentages of fibroblasts, epithelial cancer cells, and immune cells as well as the enrichment of cancer- and stroma-associated CD8^+^ T cells in longitudinally collected HGSOC samples.

## 2. Materials and Methods

### 2.1. Tissue Microarray (TMA)

**Figure 1:**
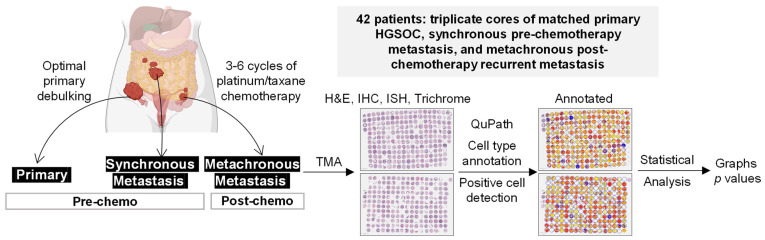
Workflow diagram of TMA generation, cell type annotation/positive cell detection, and statistical analysis. HGSOC, high-grade serous ovarian cancer; TMA, tissue microarray; H&E, hematoxylin, and eosin; IHC, immunohistochemistry; ISH, in situ hybridization.

Studies involving human tissue samples were approved by the Cedars-Sinai Institutional Review Board (IRB). We generated a TMA comprising triplicate cores of matched primary HGSOC, synchronous metastasis, and metachronous/recurrent metastasis samples from 42 patients. Primary and synchronous metastatic tumors were acquired during primary debulking surgery (pre-chemotherapy) while metachronous metastatic/recurrent tumors were acquired during second-look surgery (post-chemotherapy). Primary tumors were collected from the ovary (*n* = 38), fallopian tube (*n* = 2), and peritoneum (*n* = 2). Synchronous metastases and metachronous recurrent metastases were collected from various intraperitoneal sites, including the omentum, gastrointestinal organs, abdominal wall, liver, spleen, and lymph nodes. After a review of diagnostic H&E slides by a pathologist (AEW), the matched primary, synchronous metastatic, and metachronous recurrent HGSOC samples from 42 patients were assembled into a formalin-fixed paraffin-embedded (FFPE) TMA. Each primary, metastatic, and recurrent sample was represented by three cores (1 mm in diameter) that were punched at different locations in the tumor block (Figure 1). The 378 cores were distributed on two slides with tumor samples from 21 patients represented on each slide. The high correlation in the percentage of CD8^+^ T cells in whole slide sections and the corresponding TMA cores confirmed that the TMA cores were representative of the whole tumor section (Figure S1).

### 2.2. Immunohistochemistry (IHC), In Situ Hybridization (ISH), Masson’s Trichrome Staining, and Hematoxylin and Eosin (H&E) Staining

IHC with antibodies against alpha-smooth muscle actin (α-SMA) (α-am-1, Leica Biosystems, Wetzlar, Germany), podoplanin (PDPN) (D2-40 Cell Marque, Rocklin, CA, USA), and cluster of differentiation 8 (CD8) (JCB117, Ventana, Dallas, TX, USA) was performed by the Cedars-Sinai Medical Center Biobank and Translational Research Core using 3,3’-Diaminobenzidine (DAB) chromogen and standard protocols for automated immunostaining. The RNA hybridization kit (RNAscope 2.0 FFPE Assay) and probes for *COL11A1*, the bacterial gene *dapB* (negative control), and the housekeeping gene *HPRT* (positive control) were acquired from Advanced Cell Diagnostics, Inc. The ISH was performed according to the protocol provided by the manufacturer. Masson’s trichrome staining (hematoxylin, Biebrich scarlet/acid fuchsin, and aniline blue, Dako, Carpinteria, CA, USA) and H&E staining were performed by the Cedars-Sinai Medical Center Biobank and Translational Research Core using the Ventana Discovery XT autostainer (Roche Diagnostics, Basel, Switzerland) and standard pathology protocols.

### 2.3. Cell Type Annotation by Computational Image Analysis

H&E- and IHC-stained TMA slides were scanned at 40× magnification using the Aperio AT Turbo whole slide scanner. The image analysis was performed using QuPath software for TMA analysis (TMA DeArrayer) [14]. H&E images were used to identify and quantify fibroblasts, epithelial cancer cells, and immune cells. IHC and ISH images were used to identify and quantify subsets of fibroblasts positive for COL11A1, α-SMA, and PDPN and classify CD8^+^ cells into stroma- and cancer cell-associated CD8^+^ T cells. The workflow consisted of tissue selection (excluding folded, necrotic, and non-cancer tissues), cell/nucleus detection, annotation of regions containing three major cell types (fibroblasts, epithelial cancer cells, and immune cells), creation of the cell detection classifier using Random Trees (RTrees), application of the classifier to the TMA cores, and statistical analysis of the data (Figure 1). Cores with <200 epithelial cancer cells were excluded from statistical analyses. For multiple cores representing the same tumor, the median value was used for comparative analyses.

### 2.4. Masson’s Trichrome Image Feature Extraction and Analysis

Masson’s trichrome-stained TMA slides were scanned at 20× magnification using the Aperio AT Turbo whole slide scanner. Manual sampling was done by extracting a 200-by-200 pixels image tile of stroma adjacent to tumor from each core. Cores in which the stromal area could not be captured within the 200-by-200 pixels image tile without including cancer cells were excluded from the analysis. Image tiles were analyzed for differences in texture patterns using the blue and red channels that were digitally separated from Masson’s trichrome-stained image by a color-deconvolution algorithm [15]. Staining texture patterns were characterized by our previously applied feature panel [16,17] comprising: 1 average staining intensity, 45 segmentation-based fractal texture features (SFTA) [18], 30 Gabor mean-squared energy, and 30 Gabor mean amplitude features [19]. We chose these features because they are invariant to image rotation and can extract image intensities, stained area size, fractal dimension, and texture energy. As such, these features are applicable to many image texture quantification tasks, including computational pathology. Features were extracted separately from the blue and red stain images resulting in 212 features per tile. To characterize a tile with SFTA features, the tile undergoes a binary image decomposition into 15 binary masks and each mask is used to calculate fractal dimension, stained area, and mean intensity under the mask in the original image.

### 2.5. Identification and Validation of Computational Image Features Associated with COL11A1 Positivity

Paired digital slides with TMAs stained with Masson’s trichrome and separately with COL11A1 ISH were split into discovery and validation sets. The discovery TMA with COL11A1 staining was manually annotated for regions of interest (ROIs) with COL11A1 positive (COL11A1^+^) and COL11A1 negative (COL11A1^−^) staining, and the ROI boundaries were transferred to the corresponding digital Masson’s trichrome-stained TMA slide. Sampling was done by extracting a 200-by-200 pixels image tile from each ROI and labeled based on COL11A1 staining positivity. Image tiles (100 COL11A1^+^ and 99 COL11A1^−^) were analyzed for differences in texture patterns using the features extracted from images as described in Section 2.4. and statistical analysis as described in Section 2.6. Extraction of image tiles (*n* = 151) and features from the validation TMA stained with Masson’s trichrome was performed identically to the training set except that the COL11A1 positivity for these tiles was unknown at the time of feature extraction. To determine whether COL11A1^+^ and COL11A1^−^ regions can be predicted from Masson’s trichrome-stained slides in the validation TMA, the full set of features from the discovery TMA tiles was used to train a Random Forest (RF) classifier. The RF-based predictions of COL11A1 were then compared with the COL11A1 positivity assessed under the microscope (10× objective) in a COL11A1 ISH-stained consecutive slide.

### 2.6. Statistical Analyses

The statistical analyses were performed with SAS 9.4. In the descriptive statistics, boxplots were used to show the overall results among the three groups (primary, metastatic, and recurrent tumors). Significant variables were then analyzed using the ANOVA test. A *p*-value threshold of 0.05 was considered statistically significant. For texture pattern comparisons between primary, metastatic, and recurrent tumors, as well as between COL11A1^+^ and COL11A1^−^ regions, our null hypothesis for each analysis was that there was no difference between the groups. To test this hypothesis, the 212 features extracted from the tiles were z-scored and statistically analyzed using one-way ANOVA followed by the Tukey–Kramer test for multiple comparisons to identify differential texture features between the groups. The significance level α in the ANOVA was Bonferroni-corrected (α = 0.05/212). We rejected the null hypothesis, if at least one differentially expressed feature was found. Low-dimensional representation of the tiles characterized by differentially expressed features was visualized by the uniform manifold approximation and projection (UMAP) plot. The image analyses and visualizations were carried out in Matlab (ver. R2022a, Matlab, Natick, MA, USA). Populations of CAFs expressing COL11A1, α-SMA (ACTA2), and PDPN were visualized using t-distributed stochastic neighborhood embedding (t-SNE) plots generated by the SCope visualization tool for single-cell RNA-seq datasets (https://scope.aertslab.org/, accessed on 16 August 2022).

## 3. Results

### 3.1. Primary Tumors and Metastases Have Different Percentages of Fibroblasts, Epithelial Cancer Cells, and Immune Cells

**Figure 2:**
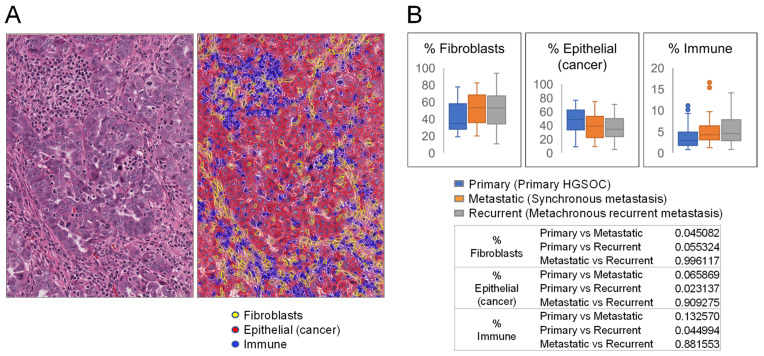
Percentages of fibroblasts, epithelial cancer cells, and immune cells in primary tumors and synchronous and metachronous metastases. (**A**) Example of H&E-stained HGSOC before and after annotation of fibroblasts, epithelial cancer cells, and immune cells by QuPath analysis. (**B**) Box and whisker plots show percentages of fibroblasts, epithelial cancer cells, and immune cells in primary HGSOC and synchronous and metachronous recurrent metastases. The *Y*-axis represents the percentage of a specific cell type among all detected cells. The table shows *p* values for comparisons between every two groups of samples within the three groups.

To study potential changes in the microenvironment during HGSOC progression, we utilized our TMA from 42 patients comprising triplicate cores of matched primary HGSOC, synchronous metastasis, and metachronous recurrent metastasis samples (hereafter referred to as primary, metastatic, and recurrent, respectively) (Figure 1). Using QuPath, we trained the object classifier to annotate the three major cell types in HGSOC (fibroblasts, epithelial cancer cells, and immune cells, Figure 2A) and quantified their percentages in TMA cores of primary, synchronous metastatic, and metachronous recurrent metastatic tumors. Compared to primary HGSOC, synchronous metastatic and metachronous recurrent metastatic HGSOC had a higher percentage of immune cells and fibroblasts but a lower percentage of epithelial cells although most of the observed differences were not statistically significant (Figure 2B). Synchronous metastatic and metachronous recurrent metastatic tumors had similar percentages of fibroblasts, epithelial cancer cells, and immune cells, suggesting that post-tumor-debulking platinum-based chemotherapy does not have an effect on the percentage of these cell types.

### 3.2. COL11A1^+^, α-SMA^+^, and PDPN^+^ CAF Subsets Show Differential Distribution during HGSOC Progression

**Figure 3:**
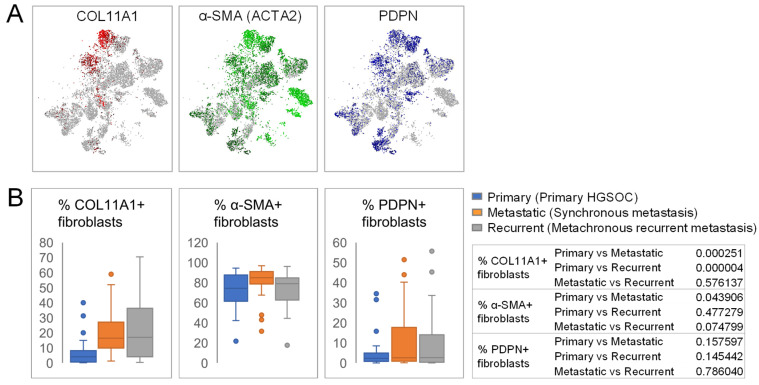
Distribution of three CAF subsets during HGSOC progression. (**A**) t-SNE plots of COL11A1, ACTA2 (encoding for α-SMA), and PDPN mRNA expression in ovarian CAFs. The images were generated using SCope software and public single-cell RNA sequencing data deposited into the ArrayExpress database at EMBL-EBI under accession numbers E-MTAB-8107. (**B**) Box and whisker plots show percentages of COL11A1^+^, α-SMA^+^, and PDPN^+^ CAFs in primary tumors, synchronous metastases, and metachronous recurrent metastases. The *Y*-axis represents the percentage of the CAF subtype among all morphologically defined CAFs. The table shows *p* values for comparisons between every two groups of samples within the three groups.

Studies in different types of cancer have demonstrated the presence of multiple CAF subtypes that might have different and even opposing roles in cancer progression [1]. In particular, COL11A1, α-SMA, and PDPN might be associated with functionally distinct subpopulations of CAFs. We have previously shown that COL11A1 is a specific marker of activated CAFs that are associated with poor survival and enriched in metastatic HGSOC [20,21]. Although PDPN^+^ CAFs have not been studied in HGSOC, they have been associated with an immunosuppressive microenvironment and/or poor prognosis in multiple cancers, including breast, lung, esophageal, and pancreatic cancers (reviewed in [22]). A t-SNE plot of the ovarian cancer single-cell RNA sequencing profile of the tumor microenvironment [23] shows that COL11A1, ACTA2 (gene encoding for α-SMA), and PDPN are expressed in different, partially overlapping, ovarian CAF subsets, with COL11A1 exhibiting the most restricted expression (Figure 3A).

We compared the percentages of COL11A1^+^, α-SMA^+^, and PDPN^+^ CAF subsets in primary tumors, synchronous metastases, and metachronous recurrent metastases. QuPath [14] was used to identify COL11A1^+^, α-SMA^+^, and PDPN^+^ CAFs by setting a threshold for the average DAB staining intensity for each cell type. The results were displayed as percentages of CAF subsets (COL11A1^+^, α-SMA^+^, or PDPN^+^) among all CAFs (defined by morphology, Figure 2A) present in a TMA core. The percentage of each CAF subtype was increased in metastases; however, the difference was most significant for COL11A1 (Figure 3B).

### 3.3. Extracellular Matrix Texture and Pattern Differ in Primary HGSOC and Metastases

**Figure 4:**
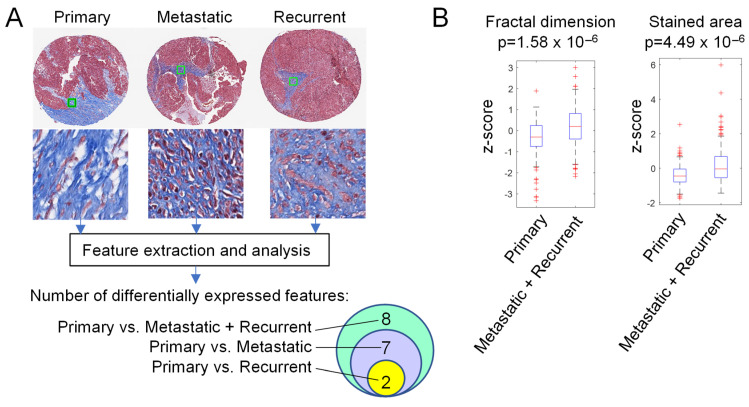
Metastatic and recurrent tumors have a different ECM texture and pattern than primary HGSOC. (**A**) Workflow diagram of tile selection for feature extraction and analysis. Shown is a randomly selected (not necessarily representative) set of TMA cores from primary, metastatic, and recurrent tumors and the corresponding manually selected stroma tiles (green squares in cores shown at higher magnification below). The number of differentially expressed features identified through statistical analysis is shown in the Venn diagram. (**B**) Examples of expression boxplots for two image features differentially expressed between Primary vs. Metastasis + Recurrence groups of image tiles.

To test for possible changes in ECM texture during HGSOC progression, representative stroma image tiles were obtained from each core in Masson’s trichrome-stained TMA slides (Figure 4A). The tiles (92 primary, 105 metastatic, 95 recurrent) were used for computational extraction of 212 image features (106 features from the blue channel and 106 features from the red channel). Statistical analysis of the 212 features revealed 8 differentially expressed features between the stroma in primary HGSOC and metastases (synchronous and metachronous combined into one group) (Figure 4A). Among these 8 features, 2 were mean blue intensity and 6 were texture features (1 fractal dimension, 1 stained area, and 4 Gabor) (Figure 4B). Seven of these 8 features were differentially expressed between primary HGSOC and synchronous metastases while 2 of the 8 features were differentially expressed between primary HGSOC and metachronous metastases (Figure 4A). None of the 212 features showed significant differential expression between synchronous metastases and matched metachronous recurrent metastases. These results suggest that collagen fibers in the ECM are different in metastases than in the primary tumors but are similar in pre- and post-treatment metastases. The identification of differentially expressed texture features (Figure 4B) also suggests that the arrangement of collagen fibers differs between primary HGSOC and metastases.

### 3.4. ECM Texture and Pattern Are Altered in Tumor Areas Positive for COL11A1

**Figure 5:**
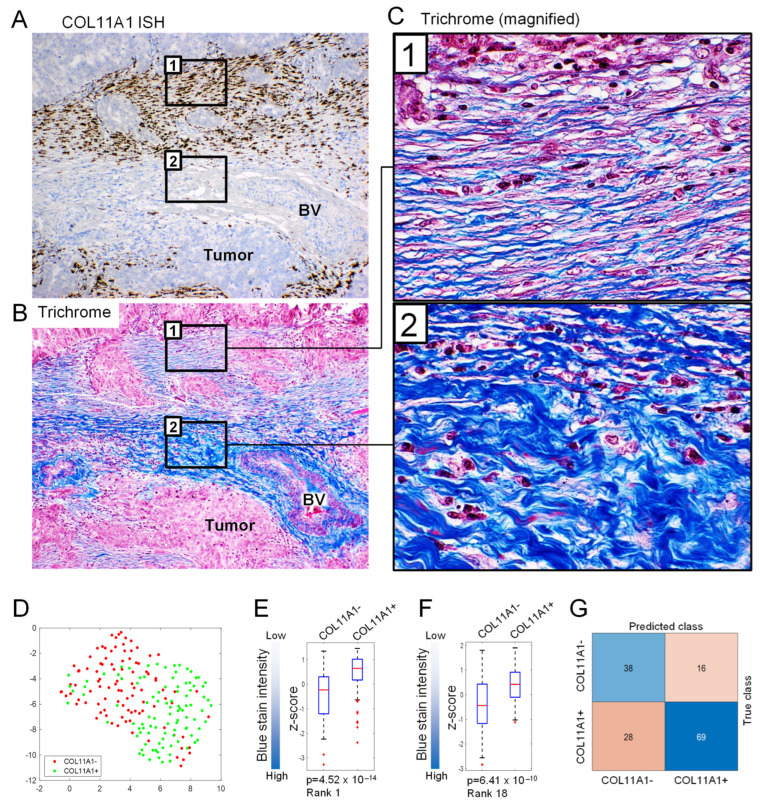
COL11A1^+^ ECM exhibits altered texture and organization. Tumor sections were processed for (**A**) COL11A1 ISH and (**B**) Masson’s trichrome staining. (**C**) COL11A1^+^ ECM (rectangles labeled [1] in (**A**,**B**)) contains thin linear collagen fibers while COL11A1^−^ ECM (rectangles labeled [2] in (**A**,**B**)) contains thick curly collagen fibers. ISH, in situ hybridization; BV, blood vessel. (**D**) UMAP plot of images characterized by the 50 differentially expressed features identified by statistical analysis of the discovery set of Masson’s trichrome tiles with known COL11A1 status. (**E**,**F**) Examples of expression boxplots for two mean blue intensity features quantitated in (**E**) image areas with strong staining (an SFTA feature) and (**F**) the entire image. (**G**) Confusion matrix showing predicted COL11A1 positivity in the validation set of Masson’s trichrome images. The classifier correctly predicted COL11A1^+^ and COL11A1^−^ scores in 107/151 images (70.86% accuracy).

Collagen type XI is primarily expressed in fetal and adult cartilage, where it assembles with collagen type II in a heterotrimeric molecule composed of α1(XI), α2(XI) and α1(II) chains, which are encoded by COL11A1, COL11A2, and COL2A1 genes, respectively [24]. Unlike most collagens, which are processed upon incorporation into the fibril structure, COL11A1 retains its N-terminal extension as a functional component throughout the lifespan of the fibril [25]. This has important implications for collagen structure and fibril diameter because the bulky N-terminal domains sterically hinder the deposition of additional collagens onto the fibril thereby preventing further increase in diameter and resulting in thin linear fibers, as in cartilage where type XI collagen helps maintain the spacing and diameter of type II collagen fibrils [26,27,28,29,30,31]. We show that this feature of thin, linear collagen fibers in the presence of COL11A1 is preserved in the tumor microenvironment. A comparison of COL11A1 ISH and Masson’s trichrome-stained TMAs revealed that COL11A1^+^ ECM frequently exhibited thin linear fibers, most of which were stained with aniline blue and Biebrich scarlet/acid fuchsin as is characteristic of newly remodeled ECM [32]. The COL11A1^−^ ECM exhibited thick curly fibers and stained primarily with aniline blue as is characteristic of mature ECM [32] (compare rectangles labeled [1] and [2] in Figure 5). Thin linear fibers have been associated with increased invasion in breast cancer [33] and increased stiffness of activated myofibroblasts in fibrosis [34].

To test in an unbiased manner if COL11A1^+^ and COL11A1^−^ tumor regions have distinct ECM patterns, we conducted computational image feature extraction and statistical analyses. Tiles from Masson’s trichrome-stained slides were divided into discovery and validation sets. Statistical analyses of 212 features extracted from the discovery tiles revealed 50 features that were differentially expressed between COL11A1^+^ and COL11A1^−^ tiles: 41 blue-channel features and 9 red-channel features (*p* < 1.66 × 10^−4^). Of the 41 blue-channel features, one was the mean blue staining intensity in the entire tile and 40 were SFTA features, which included 8 fractal features, 13 mean intensity, 11 area under the mask related features, and 8 Gabor texture features. The UMAP plot showed that COL11A1^+^ and COL11A1^−^ tiles represented by all 50 features form separate clusters (Figure 5D). Differential expressions of two of the 50 features are shown as examples in Figure 5E,F. Using the 212 features from the discovery tiles, we trained the RF classifier and applied it to classify features from the validation tiles to test whether COL11A1 status can be predicted in the validation set of Masson’s trichrome images, which were selected without prior knowledge of the COL11A1 status. Of note, during training, the RF classifier assigned a high importance score to the majority of the 50 significantly different features. Once the COL11A1 positivity of the Masson’s trichrome-stained validation tiles had been predicted by the RF classifier (54 predicted COL11A1^+^ and 97 predicted COL11A1^−^), the prediction (positive and negative) was compared to the pathology score of the entire corresponding TMA core in a consecutive COL11A1 ISH-stained slide. The pathology score of each core was determined manually using a 10× microscope objective. The COL11A1^+^ score was assigned when any COL11A1^+^ cells were observed, otherwise the TMA core was scored as COL11A1^−^. The RF’s classification performance was evaluated using a confusion matrix with juxtaposed results of the computationally predicted COL11A1 positivity in the Masson’s trichrome validation tiles and the manually determined scores from the TMA cores on the COL11A1 ISH slide (Figure 5G). The RF’s accuracy was 70.86%, and the sensitivity and specificity were 70.37% and 71.13%, respectively. Since differentially expressed features were identified between COL11A1^+^ and COL11A1^−^ regions, we rejected the null hypothesis (no difference in ECM patterns between COL11A1^+^ and COL11A1^−^ regions). This observation was supported by the accurate image classification results in the validation set.

### 3.5. HGSOC Metastases Have an Increased CD8^+^ T Cell Infiltration, However, Not All CD8^+^ T Cells Are Reaching the Tumor Parenchyma

**Figure 6:**
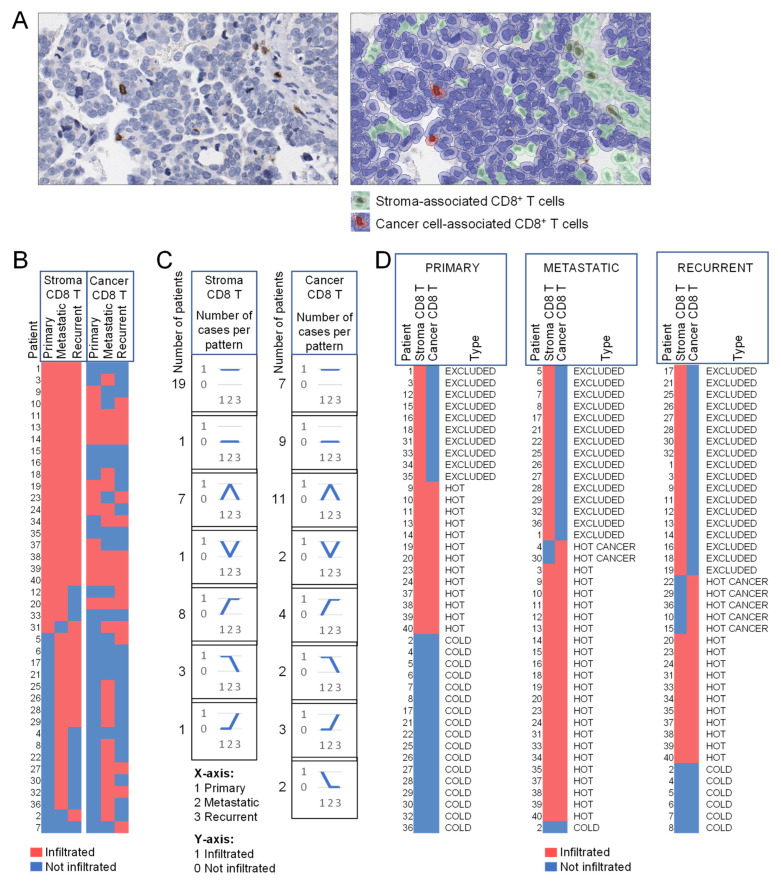
Compared to primary tumors, matched metastatic and recurrent tumors have an increased CD8^+^ T cell infiltration, however, CD8^+^ T cells are not necessarily reaching the tumor parenchyma. (**A**) Representative IHC staining with CD8 antibody (left panel) and QuPath annotation of the stroma- and cancer cell-associated CD8^+^ T cells (right panel). (**B**) Topography of stroma- and cancer cell-associated CD8^+^ T cells in matched primary, metastatic, and recurrent tumors from 40 HGSOC patients. An arbitrary threshold of 2% of CD8^+^ T cells was used to assign the ‘infiltrated’ or ‘not infiltrated’ phenotype. (**C**) Quantification of the same patients with different patterns of the ‘infiltrated’ and ‘not infiltrated’ phenotype in the stroma and cancer cell area across patient-matched primary, metastatic, and recurrent tumors. The patterns of immune infiltration are represented by diagrams. The number of patients exhibiting a specific pattern is shown on the left of each corresponding diagram. (**D**) Classification of the same patients into hot, cold, and excluded tumor phenotypes. EXCLUDED = infiltrated in stroma but not cancer, HOT = infiltrated in stroma and cancer, HOT CANCER = infiltrated in cancer but not stroma, COLD = not infiltrated.

T-lymphocyte infiltration has been linked to improved survival in multiple ovarian cancer studies [12,35,36,37,38,39,40,41,42,43,44,45]. However, most of the studies were only done on primary tumors and did not distinguish between stroma- and cancer-cell-associated CD8^+^ T cells. We annotated stroma- and cancer cell-associated CD8^+^ T cells using QuPath (Figure 6A) in a cohort of 40 patients represented by TMA cores from matched primary, metastatic, and recurrent tumors (two of the 42 patients were excluded from the analysis due to an insufficient quantity of fibroblasts or epithelial cancer cells in the cores). The median percentage of stroma- and cancer cell-associated CD8^+^ T cells in the cores from primary, metastatic, and recurrent tumors from each patient are shown in Supplementary Table S1. For simplification, an arbitrary threshold of 2% was used to assign ‘infiltrated’ (>2% CD8^+^ T cells) or ‘not infiltrated’ (<2% CD8^+^ T cells) status. The tumor stroma was infiltrated in the majority of patients, with primary tumors being less frequently infiltrated than metastases and recurrent tumors (Figure 6B). Cancer cell-associated CD8^+^ T cells in the same cores showed a more complex distribution and did not necessarily follow the same pattern of infiltration as the stroma-associated CD8^+^ T cells (Figure 6B). We quantified different patterns of CD8^+^ T cell infiltration in matched primary, metastatic, and recurrent tumors. The ‘infiltrated’ phenotype across primary, metastatic, and recurrent tumors was present in 19 of 40 patients in the stromal region but only in 7 of 40 patients in the cancer cell region (Figure 6C). In contrast, the ‘not infiltrated’ phenotype across primary, metastatic, and recurrent tumors was present in 1 of 40 patients in the stromal region and in 9 of 40 patients in the cancer cell region (Figure 6C).

Based on the distribution of immune infiltrates in the stroma and cancer cells, solid malignancies can be divided into hot (inflamed), cold (desert) and excluded (immune cells excluded from the vicinity of cancer cells) [46,47]. While hot and cold tumors are characterized by the ubiquitous presence and absence of immune cells, respectively, excluded tumors are characterized by immune cells ‘trapped’ in the stroma without access to cancer cells [46,47]. In general, patients with hot tumors have better survival than patients with cold tumors. Patients with excluded tumors also have poor survival despite the presence of immune cells in the stroma [48], suggesting that stroma-sequestered immune cells are deficient in providing immune protection against cancer cells.

Using the same definition of ‘infiltrated’ and ‘not infiltrated’ phenotype as in Figure 6C, we classified the patients into hot, cold, and excluded tumors. While all primary tumors could be assigned into relatively evenly distributed hot, cold, and excluded tumors, the synchronous and metachronous metastases were primarily excluded or hot (Figure 6C). A new category, which we designated as hot cancer (not infiltrated stroma but infiltrated cancer), appeared in synchronous and metachronous metastases (Figure 6C). Together, these data show that metastases are typically better infiltrated with CD8^+^ T cells than primary tumors but many of these infiltrates are excluded from the tumor parenchyma.

## 4. Discussion

Despite technological advances in the molecular characterization of primary ovarian cancer, the evolution of the tumor microenvironment during metastatic progression and recurrence after chemotherapy remains poorly understood. Several bulk RNA-seq studies have analyzed differences in the tumor microenvironment of longitudinally collected primary, metastatic, and/or recurrent HGSOC samples. Recent RNA-seq expression analysis of primary, metastatic, and recurrent ovarian cancer from 32 patients by Pietila et al. [49] provided detailed gene signatures of the ECM transcriptome (1027 matrisome genes defined by Naba et al. [50]). Similar to our observation that primary HGSOC are different from synchronous pre-treatment metastases and metachronous recurrent metastases in cell type composition (fibroblasts, epithelial cancer cells, and immune cells) and specific ECM proteins), Pietila et al. found the largest differences in transcriptomes between primary ovarian tumors and the matched metastases [49]. Although notable transcriptomic differences were observed between pre-and post-chemotherapy samples, the individual differentially expressed genes varied greatly depending on the anatomic location of sample collection [49] making it difficult to attribute specific changes in the matrisome to chemotherapy alone. Using RNA-seq analysis, Kreuzinger et al. [51] compared patient-matched primary and recurrent fresh-frozen tissue samples from 66 HGSOC patients. In their study, tumor microenvironment was the most significant contributor to the differentially expressed genes, which included multiple ECM genes [51]. Similar to our findings, Kreuzinger et al. found that most of the changes in the tumor microenvironment already existed in pre-treatment metastases [51]. Using the NanoString gene expression profile and IHC analyses of FFPE samples, Westergaard et al. investigated the molecular features of matched primary and recurrent HGSOC from nine patients with specific emphasis on the immune infiltrates [52]. Although the site of tumor collection was not defined in their study, based on the observed high expression of the adipocyte marker CD36 in recurrent samples, it is likely that primary tumors were obtained from the ovary/fallopian tube/adnexa while recurrent tumors were from the omentum or soft tissue in the peritoneal cavity. In the study by Westergaard et al., gene signatures of fibroblasts and immune cells were generally expressed at higher levels in recurrent tumors than in primary tumors [52]. Two additional transcriptomic studies reported increased transcriptomic signatures of the overall population of immune cells [53] and extensive ECM changes [54] in HGSOC metastases when compared to primary tumors. Thus, our computational cell morphology and IHC analyses in primary HGSOC, pre-treatment metastases, and post-treatment recurrent metastases concur with the observed differential expression of gene signatures in published transcriptomic studies.

Transcriptomic studies have shown that primary ovarian carcinomas can be classified into 4 main molecular subtypes: mesenchymal (C1), immunoreactive (C2), proliferative (C4), and differentiated (C5) [55,56,57]. Abdominal/omental metastases, however, are typically classified as the mesenchymal (C1) subtype irrespective of the subtype of patient-matched primary ovarian tumor [56,58]. The mesenchymal subtype is characterized by desmoplasia and enrichment in myofibroblasts expressing COL11A1, α-SMA, and PDPN. The mesenchymal phenotype of metastases is likely a reflection of the tumor location (ovary/adnexa in the pelvis vs. omentum/peritoneum in the upper abdomen) rather than differences between primary tumors and metastases because the majority of primary peritoneal carcinomas (PPC) are classified as the mesenchymal subtype [59] and there is evidence that mesenchymal primary tumors may actually be PPC metastases to the ovary [60] or secondary metastases to the ovary [61,62]. Together, these results suggest that the mesenchymal phenotype is determined by the metastatic microenvironment rather than by the intrinsic molecular subtype of epithelial cancer cells.

Notably, bulk RNA-seq analyses lack the spatial information necessary to attribute the differentially expressed genes to specific cell types and ECM organization. Our quantitation of the percentage of CAFs expressing extracellular matrix components COL11A1, α-SMA, and PDPN revealed that COL11A1^+^ fibroblasts were increased in both synchronous and metachronous recurrent metastases compared to matched primary HGSOC. We and others have shown that COL11A1 expression in primary HGSOC is associated with adverse clinical outcomes, including late-stage diagnosis, residual disease after primary cytoreduction, chemotherapy resistance, higher rate of metastasis and recurrence, and poor overall survival [20,21,63,64,65,66,67]. While it remains unclear how COL11A1 might contribute to the poor outcomes in ovarian cancer, we observed that areas enriched for COL11A1 are associated with altered ECM texture and pattern, including fiber linearization. In breast cancer, collagen fibers aligned into linear tracks at the cancer-stroma interface has been associated with an increased incidence of metastasis [33,68,69,70,71], suggesting that such changes in collagen organization might facilitate chemotaxis of cancer cells along the linear collagen tracks. Although evidence is currently lacking, it is also possible that COL11A1-rich ECM is less penetrable to chemotherapy and immunotherapy.

Collagen structure in ovarian cancers has been analyzed by two-photon excited fluorescence (TPF) and second harmonic generation (SHG) imaging [72]. A comparison of normal peritoneal biopsies and matched peritoneal metastases revealed that collagen fibers in metastases were thinner than their normal tissue counterparts [73]. Through the analysis of tissue images, we found that COL11A1 expression is associated with distinct ECM architecture in Masson’s trichrome-stained slides (thin, linear collagen fibers with reduced aniline blue staining). By applying a trained classifier to random tiles extracted from TMA cores of Masson’s trichrome-stained slides, we were able to predict which TMA cores were COL11A1^+^. Although the classifier is not sufficiently accurate to use in a clinical setting, it provides evidence that the expression of a gene associated with poor outcomes in ovarian cancer can be predicted based on ECM architecture.

## 5. Conclusions

Our computational image analyses of primary HGSOC and patient-matched pre-treatment and post-treatment peritoneal metastases show differences between primary HGSOC and synchronous and/or metachronous metastases in: (1) percentages of fibroblasts, epithelial cells, and immune cells, (2) percentages of COL11A1^+^ fibroblasts, (3) ECM architecture, and (4) percentages of stroma- and cancer-associated CD8^+^ T cells. Pre-treatment synchronous metastases and post-treatment metachronous metastases did not differ significantly in their ECM composition and architecture, suggesting that the observed molecular and structural differences between primary and recurrent HGSOC can be largely attributed to the tumor location rather than the effects of treatment on the host microenvironment that subsequently contributes to tumor recurrence.

## Data Availability

The raw and processed data presented in this study are available upon request from the corresponding author.

## Supplementary Materials

The following supporting information can be downloaded at: https://www.mdpi.com/article/10.3390/cells11233769/s1, Figure S1: Correlation between % of CD8-positive immune cells in whole slides vs. TMA cores; Table S1: Forty patients with high-grade serous ovarian cancer (HGSOC) were analyzed for the median percentage of stroma- and cancer cell-associated CD8-positive cells using QuPath.
