# Supplementary material for: Dynamic Changes in the Extracellular Matrix in Primary, Metastatic, and Recurrent Ovarian Cancers
Source: Cells. 2022 Nov 25;11(23):3769. doi: 10.3390/cells11233769 (PMC9736731; doi:10.3390/cells11233769)
Supplement: Supplementary file 1 [file cells-11-03769-s001.zip › cells-2013159-supplementary.pdf]

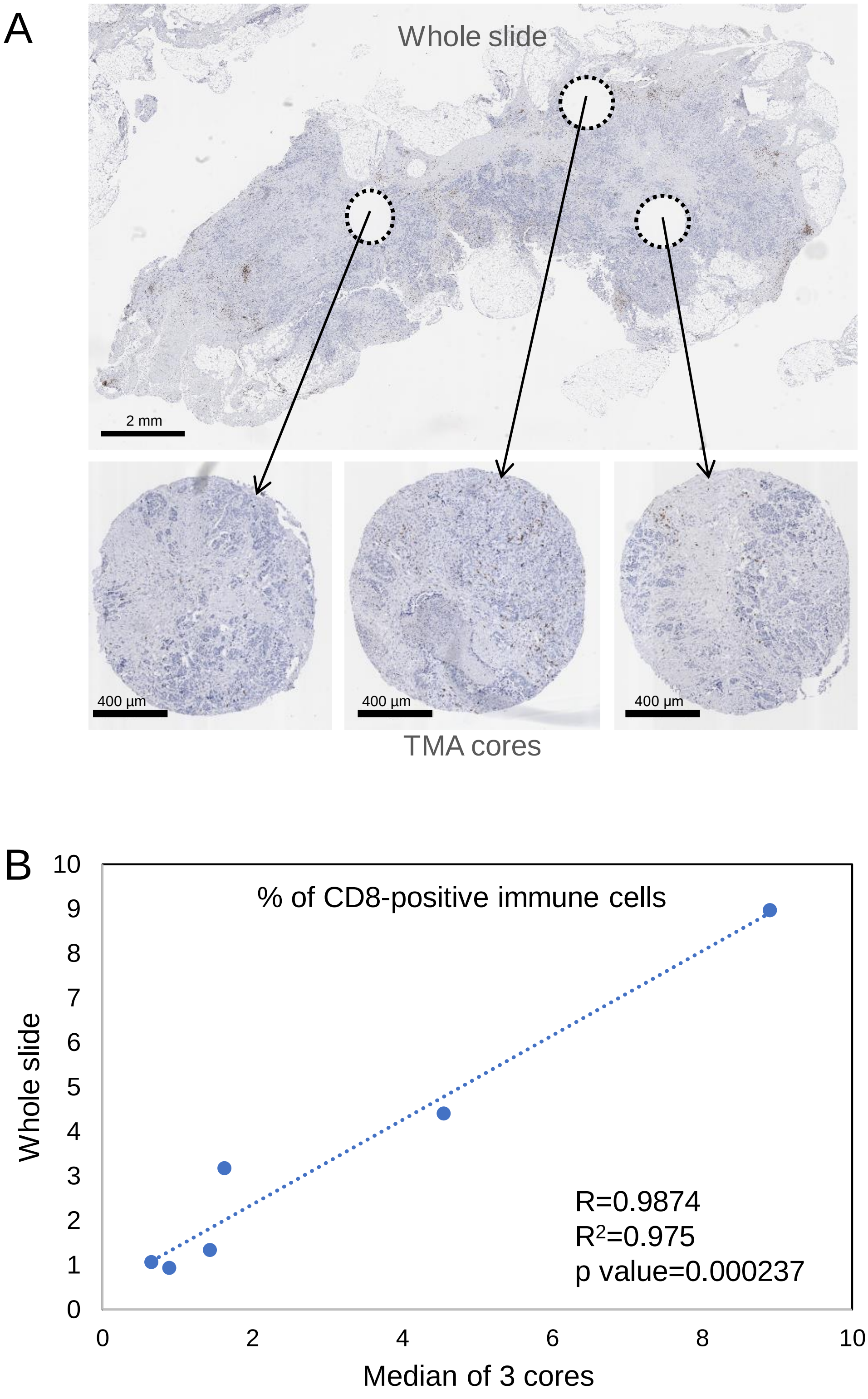

Supplementary Figure S1. Correlation between % of CD8-positive immune cells in whole slides vs TMA cores. **(A)** A whole section and the corresponding TMA cores stained with CD8 antibody with a hematoxylin background. **(B)** Pearson correlation between % CD8-positive immune cells in 6 whole sections and the median of 3 corresponding TMA cores from each section. CD8-positive immune cells were identified using QuPath.

Patient	% stroma-associated CD8 T			% cancer cell-associated CD8 T		
	Primary	Metastatic	Recurrent	Primary	Metastatic	Recurrent
1	11.97	4.225	27.78	2.443	1.384	26.07
2	1.383	1.006	3.4055	0.2124	0.2816	1.31905
3	3.054	5.939	2.143	2.079	2.22	0.8403
4	1.151	3.991	0.9674	0.1928	1.674	0.0509
5	0.3879	17.93	2.983	0.4938	4.014	0.6327
6	1.169	2.763	2.206	1.3029	1.174	0.6148
7	1.439	1.688	1.807	0.5688	0.5319	0.545
8	0.8652	8.491	1.656	0.0681	6.249	0.1835
9	3.417	7.833	5.163	3.312	2.89	2.256
10	12.2	12.255	15.12	5.373	6.1535	19.13
11	12.04	8.358	31.535	6.644	2.444	15.105
12	2.121	6.25	0.3183	0.6092	1.74	0.0435
13	2.873	7.844	2.305	3.527	1.2317	0.7192
14	11.566	6.884	3.775	6.349	3.419	2.7365
15	3.686	5.14	9.165	1.814	3.994	2.73
16	12.516	6.389	8.428	1.746	3.8565	1.571
17	0.2746	4.907	4.95	0.0355	0.7697	1.021
18	4.049	6.625	8.699	1.006	3.612	3.22
19	11.87	12.63	6.596	7.013	5.867	2.677
20	9.568	3.237	1.882	5.816	0.8576	2.541
21	1.241	3.57	17	0.3384	0.3795	5.733
22	0.3243	11.74	0.2614	0.1332	3.757	0.14795
23	6.767	13.86	3.11455	1.491	6.333	2.64295
24	4.4435	2.847	6.055	1.091	0.1395	0.8943
25	1.627	7.109	11.19	0.4441	0.4184	3.681
26	0.36495	5.71	2.95965	0.01165	2.513	0.1117
27	0.7678	21.85	0.82005	0.0543	6.075	0.35515
28	0.5079	19.71	4.508	0.119	6.488	0.9434
29	1.821	5.259	27.46	0.2805	0.8054	11.5
30	0.6315	6.425	1.63	0.1145	1.159	0.172
31	3.152	1.294	6.314	0.7531	0.1416	1.036
32	0.3551	10.2	1.806	0.0655	2.255	0.0494
33	20.735	4.472	1.962	7.0385	2.386	0.2421
34	6.1095	2.959	2.874	2.302	1.612	0
35	2.15	10.88	5.473	0.21665	5.738	1.565
36	1.598	7.139	1.321	0.202	9.463	0.0728
37	6.333	20.735	5.104	1.37	7.546	3.511
38	22.63	18.57	6.858	33.06	13.11	4.088
39	4.403	9.7885	14.46	0.9833	6.9625	0.6649
40	5.928	6.905	10.19	2.049	3.925	4.139

Supplementary Table S1. Forty patients with high-grade serous ovarian cancer (HGSOC) were analyzed for the median percentage of stroma- and cancer cell-associated CD8-positive cells using QuPath. The numerical values are color coded for easier visualization of CD8-positive cell patterns in each patient.
